# Spatial analysis of residential location at birth, PFAS in public water, and childhood cancers in Southern California (2000–2019)

**DOI:** 10.1038/s41370-026-00850-1

**Published:** 2026-03-05

**Authors:** Natalie R. Binczewski, Libby M. Morimoto, Joseph L. Wiemels, David B. Richardson, Scott M. Bartell, Catherine Metayer, Veronica M. Vieira

**Affiliations:** 1 Department of Environmental and Occupational Health, Joe C. Wen School of Population & Public Health, University of California, Irvine, CA USA; 2 Division of Epidemiology, School of Public Health, University of California, Berkeley, CA USA; 3 Center for Genetic Epidemiology, Department of Population and Public Health Sciences, University of Southern California Keck School of Medicine, Los Angeles, CA USA

## Abstract

**Background:**

Perfluoroalkyl substances exposure via drinking water varies spatially and may be associated with childhood cancer incidence.

**Objective:**

We examined associations between residence at birth and incidence of childhood cancer in Orange and Los Angeles Counties, California, and if these associations change after adjustment for perfluorooctanesulfonic acid (PFOS) and perfluorooctanoic acid (PFOA) detection in public water supplies and other maternal characteristics.

**Methods:**

Cancer cases (*n* = 6448) diagnosed before age 22 years between 2000 and 2019 and controls (*n* = 13,044) were categorized by PFOS/PFOA detection in residential public water according to the US Environmental Protection Agency third Unregulated Contaminant Monitoring Rule (UCMR3). Referent models using generalized additive models included a bivariable smoothing for location, child birth year, age at diagnosis or index year, and sex. Spatial models were additionally adjusted for PFOS/PFOA detection and maternal characteristics. We calculated odds ratios (ORs) and 95% confidence intervals (CIs) for the effect of location and PFOS/PFOA detection. We also stratified analyses by US-born and Mexico-born mothers.

**Results:**

Locations with significant increased risk of all cancers, leukemias, lymphomas, and other solid tumors were collocated with PFOS/PFOA contaminated water supplies. Referent ORs for location were attenuated after adjusting for PFOS detection and in stratified analyses for children of US-born mothers but not for Mexico-born mothers. PFOS detection was associated with higher odds of neuroblastoma (OR = 1.51, 95% CI: 1.07, 2.13) and retinoblastoma (OR = 1.83, 95% CI: 1.19, 2.79), whereas all cancers (OR = 1.07; 95% CI: 0.97, 1.18), brain tumors (OR = 1.15, 95% CI: 0.96, 1.37), and other solid tumors (OR = 1.12, 95% CI: 0.98, 1.29) had non-significant associations. PFOA detection was associated with elevated risk of retinoblastoma (OR = 1.85, 95% CI: 1.14, 3.01).

**Significance:**

PFOS and PFOA were associated with higher childhood cancer incidence; however, their detection in drinking water did not fully explain observed geographic variations in risk.

**Impact:**

In our population-based study of childhood cancers in Orange and Los Angeles Counties, California, we observed increased risks of all cancers combined and some cancer subtypes (i.e., brain tumors, leukemia, neuroblastoma, and retinoblastoma) among children with birth addresses located in public water systems with PFOS/PFOA contamination. Our results suggest that prenatal PFOS/PFOA exposure may explain some of the cancer risk across the study area, and the effects of PFOS/PFOA adjustment on the spatial patterns of cancer risk were stronger among children of US-born mothers. This work can inform policies to reduce exposure to perfluoroalkyl substances and reduce possible health impacts.

## Introduction

Environmental exposures are often explored as potential explanations for cancer clusters, a greater-than-expected number of cancer cases in a population in a specific place and time [1, 2]. Epidemiologic studies have documented associations between cancer and several environmental factors, including radiation, air pollution, and pesticides and childhood cancer risk [3–8]. Several childhood cancer analyses have found positive associations between contaminated water in Massachusetts [9] and vector-borne disease in Nevada [10] with childhood leukemia clusters. Additionally, clusters of leukemia, brain tumors, and rhabdomyosarcomas were found in areas near areas with per- and polyfluoroalkyl substances (PFAS) contamination [11–13]. The current study was motivated by a previous analysis of California childhood cancers that found spatial-temporal clustering of acute lymphoblastic leukemia (ALL) in Northern California and malignant gonadal germ cell tumors in Southern California [14], however environmental exposures were not considered as potential risk factors in that study.

PFAS are environmental contaminants linked to a variety of adverse health effects, including cancer in both animal and human studies [15, 16]. Water consumption is thought to be a major pathway for PFAS exposure in adults, and PFAS can be transferred to infants during pregnancy and breastfeeding [17, 18]. Various point sources, such as industrial plants, wastewater treatment plants, landfills, and firefighting training sites have released PFAS into the environment [19]. As part of the Safe Drinking Water Act, most public water systems in the United States were required to monitor for a set of unregulated contaminants, including 6 PFAS chemicals during 2013 to 2015 under the US Environmental Protection Agency third Unregulated Contaminant Monitoring Rule (UCMR3) [20]. 26 California water systems had detectable PFAS reported in the UCMR3 data; most of the affected water systems were located in Los Angeles and Orange Counties. More recently, in the fifth Unregulated Contaminant Monitoring Rule (UCMR5), 29 of the 30 contaminants required to be monitored were in the PFAS family of chemicals [21]. The UCMR5 data collection and reporting is not yet complete and will continue from 2023 through 2025.

Recent work has studied associations between drinking water contamination and incident cancers using UCMR3 and UCMR5 PFAS data. Using county-level cancer incidence data from 2016 to 2021, increased incidences of digestive, endocrine, oral cavity, and respiratory cancers were associated with PFAS exposure across the United States [22]. Binary variables for detection of or violation of Maximum Contaminant Levels were created and used as the PFAS exposure variables in the analysis while adjusting for other county-level covariates [22]. In our prior analysis of childhood cancers, we estimated maternal serum concentrations of perfluorooctanesulfonic acid (PFOS) and perfluorooctanoic acid (PFOA) based on UCMR3 data for California and adjusted for individual-level maternal characteristics [23]. We found suggestive evidence that PFOS or PFOA exposures through drinking water were associated with an increased risk of various childhood cancer types, including acute myeloid leukemia and Wilms tumors [23].

The aim of this case-control study was to explore spatial clustering of childhood cancer cases in Los Angeles County and Orange County, California. We used generalized additive models (GAMs) to model the effect of individual-level birth location on the risk of childhood cancers and to determine if adjustment for PFAS in drinking water reported during UCMR3 monitoring and other individual-level socio-demographic characteristics contributed to the observed geographic patterns.

## Methods

### Study population and design

Our study population included children born between 2000 and 2019 who had a birth address in a Los Angeles or Orange County zip code (Supplemental Fig. 1A). A case-control design was used in which cancer cases were diagnosed through December 2021. Controls from the California Linkage Study of Early-onset Cancers (CALSEC) were children in the study population who were cancer-free through 2021. The overall study design linking the California cancer and birth registries and data collection for CALSEC has been described in previous literature [24–26]. Cases (*n* = 6554) and controls (*n* = 13,046) were frequency-matched (1:2) on birth year. For cases with multiple primary cancer diagnoses (*n* = 104), only the first diagnosis was retained.

Cases and controls were geocoded using the StreetMap Premium USA locator in ArcGIS Pro version 3.2.0 (ESRI, Redlands). A total of 19,396 birth addresses (99%) were geocoded to the street address level, with the remaining geocoded to street names (*n* = 63) or zip codes (*n* = 37). We further excluded four cases and controls that were not located in the contiguous Los Angeles or Orange County area (e.g., Catalina Island).

Cancer cases were classified according to the International Classification of Childhood Cancer (ICCC) site recode (Supplemental Table 1). We examined all-cancers, leukemias, lymphomas, brain tumors, and other solid tumors, as well as specific subtypes that had suggestive associations with PFAS exposure in previous literature: neuroblastoma, nephroblastoma, non-Hodgkin lymphoma, retinoblastoma, lymphoid leukemias and acute myeloid leukemia [23, 27, 28]. The study was reviewed and approved by the institutional review boards at the California Department of Public Health, the University of California, Berkeley, and the University of California, Irvine.

### PFOS/PFOA in public water

The UCMR3 included PFAS monitoring data for 123 water systems serving Los Angeles and Orange County zip codes, with 17 of these water systems having reportable levels of PFOS or PFOA between 2013 and 2015 (Supplemental Fig. 1B). The primary source of PFAS contamination in the study area drinking water is waste water recharge processes, and data from the local water districts indicates that PFAS contamination remained detectable after UCMR3 monitoring concluded in 2015 until contaminated wells were taken off-line in 2019 [29]. For each of the 17 water systems, binary variables for detection of PFOS and/or PFOA in water [22] were assigned to a water district if there were analytical detection values above minimum reporting levels included in the UCMR3 data (0.04 µg/L for PFOS, 0.02 µg/L for PFOA). PFAS water contamination data were linked to a shapefile downloaded from the California State Geoportal [30] with service area boundaries of public water systems.

Maternal residences at birth during the years 2000 to 2019 were joined to corresponding water district boundaries using ArcGIS Pro version 3.2.0 (ESRI, Redlands). Children with birth addresses outside of water district service boundaries with reported PFOS/PFOA in UCMR3 monitoring data were assigned as non-detect for PFOS/PFOA exposures through drinking water. PFOA detects ranged from 0.021 to 0.027 µg/L (median = 0.024 µg/L) and PFOS detects ranged from 0.042 to 0.053 µg/L (median = 0.050 µg/L). Because PFOS or PFOA concentrations below the minimum reporting limits were very common and would not be captured by use of continuous exposure measures, we chose to use “non-detect” and “detect” as our exposure measure, similar to previous work [22, 31].

### Statistical analysis

GAMs with bivariable smoothing for individual location were used to estimate the log odds of childhood cancer across the study area. In addition to the bivariable smooth term, these semi-parametric models include linear covariates which allows for calculation of effect estimates for other risk factors associated with childhood cancers. The optimal span of the smooth was calculated for each model by minimizing the Akaike information criterion (AIC). For comparisons of regional variation between cancer groups, we also used an a priori span of 0.20 for all models [32, 33]. Analyses were performed using the MapGAM package in R version 4.2.2 (R Project for Statistical Computer, Vienna, Austria).

GAMs followed the form of Eq. 1, where the left-hand side is the logarithm of disease odds at a given location (X,Y) and α is a vector of parameters associated with Z, a vector of covariates. The predicted log odds at each X,Y point was divided by the median log odds across a grid of 3529 rectangles approximately 1.2 km in the X direction by 1.5 km in the Y direction covering the study area to calculate odds ratios (OR); as such, the range of ORs for each map must include values below and above one. The ORs were then mapped to present the predicted ORs for childhood cancers associated with location throughout the study area. ORs represent the association between location and childhood cancers predicted at the median value for the other continuous covariates and at the value for the highest proportion of the categorical covariates. We limited the resulting maps to the most populated areas of Los Angeles and Orange Counties, similar to previous work [34]. Standard errors were used to calculate 95% confidence intervals and determine areas of statistically significant increased and decreased risk, which were displayed on the maps using black contour lines for α=0.05. The null hypothesis is that cancer incidence is not a function of location, and we present the approximate p-value for this test based on the assumption of a chi-square distribution for the difference in deviances.

1 $${{{\rm{logit}}}}\left[p\left(X,Y\right)\right]=S\left(X,Y\right)+{aZ}$$

Spatial analyses are effective for distinguishing the contributions of environmental and social risk factors to geographic variations of disease. We analyzed spatial patterns of childhood cancer using the following models: Model 1, a referent model with the smooth term for location and individual-level child characteristics (sex, continuous age, and continuous birth year); Model 2A, an exposure adjusted model that includes the binary PFOS exposure measure along with the covariates in the referent model; Model 2B, a fully adjusted model that adjusts for individual-level maternal characteristics along with covariates in Model 2A; Model 3A, an exposure adjusted model that includes the binary PFOA exposure measure along with the covariates in the referent model; and Model 3B, a fully adjusted model that adjusts for individual-level maternal characteristics along with covariates in Model 3A. Referent Model 1 represents the underlying geographic variation in childhood cancers without accounting for any environmental or maternal demographic confounders. Exposure adjusted Models 2A, 2B, 3A, and 3B further adjusted for PFOS exposure (yes or no) or PFOA exposure (yes or no) to determine whether PFOS/PFOA exposure accounted for any of the underlying spatial patterns and estimate their independent association with childhood cancers. Because PFOS and PFOA detections in drinking water were correlated (*r* = 0.53), we did not include them together in a single exposure model. Fully adjusted Models 2B and 3B adjusted for maternal characteristics of age (continuous), education (8th grade or less, 9–12th grade, at least some college, or unknown), birthplace (US, Mexico, Other), race and ethnicity (non-Hispanic white, Hispanic, Asian American Pacific Islander, and other), and insurance provider (private, public, other), in addition to the previous covariates. These additional risk factors are known to vary spatially and may provide additional information about what predictors are driving the underlying geographic patterns of childhood cancers.

Analyses stratified by maternal birthplace country were conducted for all cancers, as well as larger groups including leukemia, lymphoma, brain tumors, other solid tumors, lymphoid leukemias, and acute myeloid leukemia. The analyses for US-born mothers (3362 cases and 6338 controls) and Mexico-born mothers (1,687 cases and 3,707 controls) were motivated by differences seen in prior studies regarding childhood cancer risks in California [23, 35].

The statistical significance of the location term in each model is determined by the global *p*-value (≤0.05). The AIC for each model is presented to show goodness of fit of each model. We then analyze the impact of PFOS or PFOA on these spatial patterns by comparing referent and adjusted maps (presented with a common OR range), as well as by extracting beta-coefficients from the linear term of the GAM and calculating the ORs and 95% confidence intervals associated with PFOS/PFOA.

## Results

**Fig. 1:**
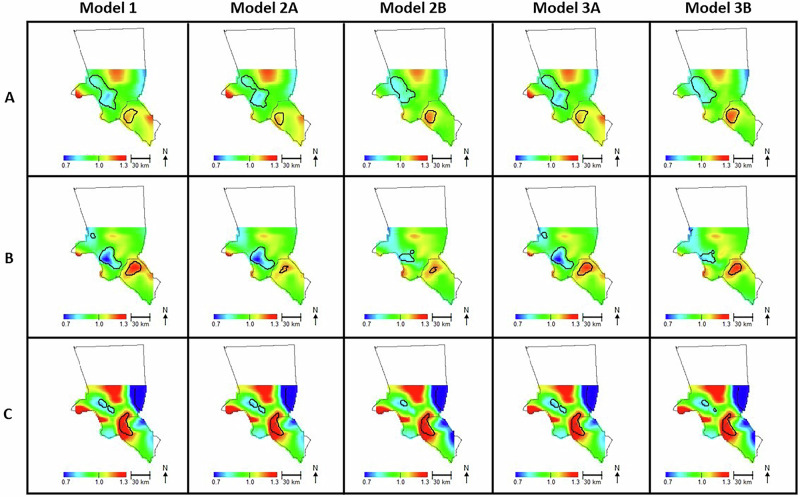
Geographic patterns of all childhood cancers in Los Angeles and Orange Counties, California, 2000-2021. Areas of statistically significant increased or decreased risk are outlined in black. **A** Models for all childhood cancer cases (*n* = 6448) and controls (*n* = 13,044). **B** Models for childhood cancer cases (*n* = 3362) and controls (*n* = 6338) born to US-born mothers. **C** Models for childhood cancer cases (*n* = 1687) and controls (*n* = 3707) born to Mexico (MX)-born mothers. Model 1 adjusts for location, child sex, child age, and child’s birth year. Model 2 A adjusts for PFOS exposure, in addition to adjustments in Model 1. Model 2B adjusts for maternal age, maternal, maternal birthplace, maternal race and ethnicity, and insurance provider, in addition to adjustments in Model 2 A. Model 3 A adjusts for PFOA exposure, in addition to adjustments in Model 1. Model 3B adjusts for maternal age, maternal, maternal birthplace, maternal race and ethnicity, and insurance provider, in addition to adjustments in Model 3 A.

**Fig. 2:**
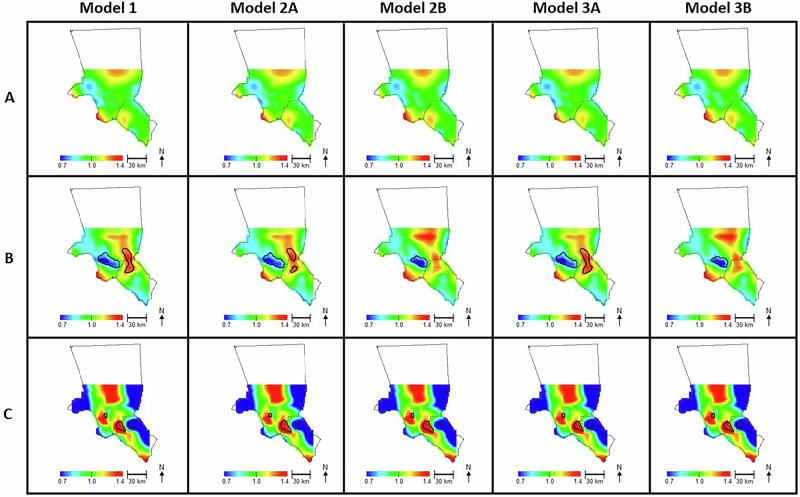
Geographic patterns of all leukemias in Los Angeles and Orange Counties, California, 2000–2021. Areas of statistically significant increased or decreased risk are outlined in black. **A** Models for leukemia cases (*n* = 2707) and controls (*n* = 13,044). **B** Models for leukemia cases (*n* = 603) and controls (*n* = 6338) born to US-born mothers. **C** Models for leukemia cases (*n* = 1028) and controls (*n* = 3707) born to Mexico (MX)-born mothers. Model 1 adjusts for location, child sex, child age, and child’s birth year. Model 2 A adjusts for PFOS exposure, in addition to adjustments in Model 1. Model 2B adjusts for maternal age, maternal, maternal birthplace, maternal race and ethnicity, and insurance provider, in addition to adjustments in Model 2 A. Model 3 A adjusts for PFOA exposure, in addition to adjustments in Model 1. Model 3B adjusts for maternal age, maternal, maternal birthplace, maternal race and ethnicity, and insurance provider, in addition to adjustments in Model 3 A.

**Fig. 3:**
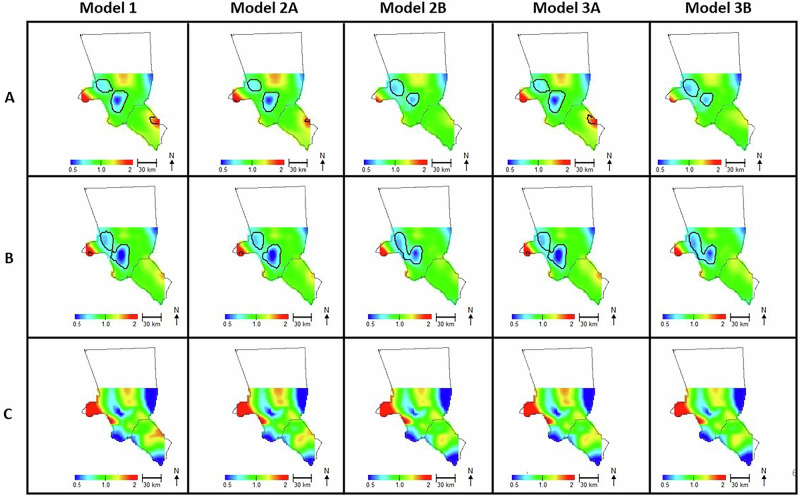
Geographic patterns of all brain tumors in Los Angeles and Orange Counties, California, 2000–2021. Areas of statistically significant increased or decreased risk are outlined in black. **A** Models for brain cancer cases (*n* = 1301) and controls (*n* = 13,044). **B** Models for brain cancer cases (*n* = 748) and controls (*n* = 6338) born to US-born mothers. **C** Models for brain cancer cases (*n* = 282) and controls (*n* = 3707) born to Mexico (MX)-born mothers. Model 1 adjusts for location, child sex, child age, and child’s birth year. Model 2 A adjusts for PFOS exposure, in addition to adjustments in Model 1. Model 2B adjusts for maternal age, maternal, maternal birthplace, maternal race and ethnicity, and insurance provider, in addition to adjustments in Model 2 A. Model 3 A adjusts for PFOA exposure, in addition to adjustments in Model 1. Model 3B adjusts for maternal age, maternal, maternal birthplace, maternal race and ethnicity, and insurance provider, in addition to adjustments in Model 3 A.

**Fig. 4:**
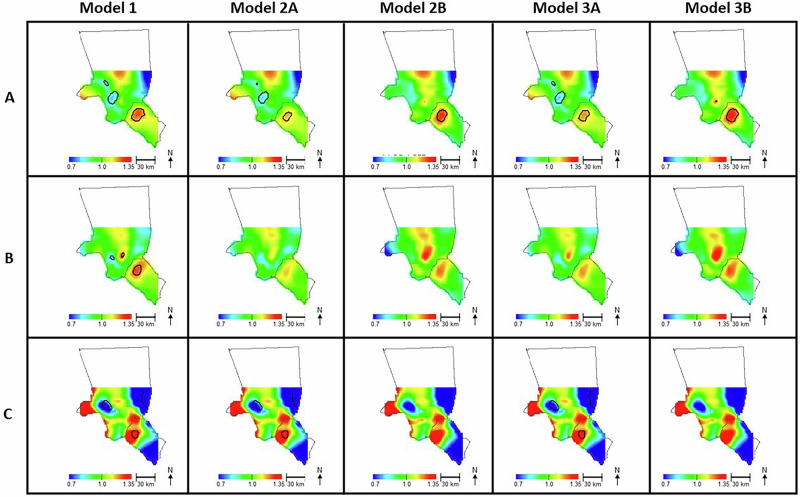
Geographic patterns of other solid tumors in Los Angeles and Orange Counties, California, 2000–2021. Areas of statistically significant increased or decreased risk are outlined in black. **A** Models for other solid tumor cases (*n* = 2341) and controls (*n* = 13,044). **B** Models for other solid tumor cases (*n* = 1197) and controls (*n* = 6338) born to US-born mothers. **C** Models for other solid tumor cases (*n* = 611) and controls (*n* = 3707) born to Mexico (MX)-born mothers. Model 1 adjusts for location, child sex, child age, and child’s birth year. Model 2 A adjusts for PFOS exposure, in addition to adjustments in Model 1. Model 2B adjusts for maternal age, maternal, maternal birthplace, maternal race and ethnicity, and insurance provider, in addition to adjustments in Model 2 A. Model 3 A adjusts for PFOA exposure, in addition to adjustments in Model 1. Model 3B adjusts for maternal age, maternal, maternal birthplace, maternal race and ethnicity, and insurance provider, in addition to adjustments in Model 3 A.

Cases and controls had similar distributions of maternal age and birth year (matching factor). However, the mothers of cases were more frequently Non-Hispanic White, US-born, have some college education, and used private insurance as compared to mothers of controls (Table 1). Case children were also more frequently male, born in Orange County, and born at a residence served by public water containing PFOS and PFOA than controls. Specifically, 12.31% (*n* = 794) of cases and 10.63% (*n* = 1387) of controls were assigned exposure to PFOS, while 7.29% (*n* = 470) of cases and 6.45% (*n* = 841) of controls were assigned exposure to PFOA through contaminated drinking water systems. In our LA and Orange County population, spatial risk patterns of childhood cancer varied across cancer groups and within cancer groups. Resulting maps are presented with a common OR range for comparison within cancer subtypes with exact predicted ranges reported in Supplemental Table 2. We present analyses with local spatial variation that differs with adjustment for PFOS/PFOA or maternal characteristics in the main text, and those with consistent geographic patterns across models are included in the Supplement. Geographic patterns of odds ratios for location associated with selected cancer types are mapped in Figs. 1–4 and Supplemental Figs. 2–5.

**Table 1:** Characteristics of study participants born in Los Angeles or Orange Counties, 2000–2019.

Characteristics	*n* cases	% cases	*n* controls	% controls	*P*-value^a^
Child’s Birth Year					1
2000–2004	2780	43.11	5636	43.21	
2005–2009	1854	28.75	3758	28.81	
2010–2014	1208	18.73	2436	18.68	
2015–2019	606	9.40	1214	9.31	
Child’s Sex					< 0.001
Female	2954	45.81	6365	48.80	
Male	3494	54.19	6679	51.20	
Child’s Birth County					< 0.001
Los Angeles County	4770	73.98	10136	77.71	
Orange County	1678	26.02	2908	22.29	
Maternal Age					0.23
< 20	479	7.43	1091	8.36	
20–24	1205	18.69	2669	20.46	
25–29	1662	25.78	3446	26.42	
30–34	1770	27.45	3354	25.71	
35–39	1032	16.00	1950	14.95	
40+ or unknown	300	4.65	534	4.09	
Maternal Education					< 0.001
8th grade or less	581	9.01	1381	10.59	
9-12th grade	2718	42.15	5709	43.77	
at least some college	3025	46.91	5729	43.92	
Unknown	124	1.92	225	1.72	
Medical Insurance					< 0.001
Private	3340	51.80	6041	46.31	
Medicare or Medi-Cal	2840	44.04	6294	48.25	
Other/Unknown	268	4.16	709	5.44	
Maternal Race and Ethnicity					< 0.001
Non-Hispanic White	1622	25.16	2755	21.12	
Hispanic	3741	58.02	7658	58.71	
Asian American and Pacific Islander	694	10.76	1668	12.79	
Other	391	6.06	963	7.38	
Maternal Birthplace					< 0.001
Other countries/Unknown	1399	21.70	2999	22.99	
US 50 States	3362	52.14	6338	48.59	
Mexico	1687	26.16	3707	28.42	
PFOS in Drinking Water					< 0.001
Exposed	794	12.31	1387	10.63	
Unexposed	5654	87.69	11657	89.37	
PFOA in Drinking Water					0.029
Exposed	470	7.29	841	6.45	
Unexposed	5978	92.71	12203	93.55	

Abbreviations: Number (*n*) and percent (%).

^a^*P*-value from chi-square test.

### Associations between PFOS/PFOA and childhood cancers

The association between location and childhood cancers among all participants varied by cancer subtype, and for some, was explained in part by PFOS/PFOA exposure and maternal socioeconomical characteristics. In referent models of location and child characteristics (Model 1), child’s birth residence was statistically significantly associated with all cancers, lymphomas, non-Hodgkin lymphoma, brain tumors, and other solid tumors, but not with all leukemias, lymphoid leukemias, acute myeloid leukemia, neuroblastoma, retinoblastoma, and nephroblastoma (Supplemental Table 3). In all models for all cancers, lymphomas, non-Hodgkin lymphoma, and other solid tumors, maps showed an area of significant increased risk in Orange County where water districts with reported levels of PFOS or PFOA were located (Supplemental Table 4**)**. Los Angeles water districts with reported PFOS/PFOA were collocated with areas of significant decreased risk in referent models of all cancers and brain tumors, and areas of significant increased risk for non-Hodgkin lymphoma, however risk in these areas was no longer elevated in fully adjusted models (Models 2B and 3B) for all cancers and non-Hodgkin lymphoma. Areas of increased risk were attenuated in size when spatial analyses were adjusted for PFOS as compared to the referent models for all cancers (Fig. 1A), all brain tumors (Fig. 3A), and all solid tumors (Fig. 4A). Similar geographic patterns of risk were observed in PFOS-adjusted (Model 2 A) and referent models for all leukemias (Fig. 2A), lymphoid leukemias (Supplemental Fig. 2A), all lymphomas (Supplemental Fig. 4A), non-Hodgkins lymphoma (Supplemental Fig. 4D), acute myeloid leukemia (Supplemental Fig. 3A), neuroblastoma (Supplemental Fig. 5A), retinoblastoma (Supplemental Figure 5B), and nephroblastoma (Supplemental Figure 5C). Areas of increased risk were attenuated in fully adjusted models for brain tumors (Fig. 3A) and lymphomas (Supplemental Fig. 4A) compared to exposure adjusted Models 2A and 3A. Adjustment for PFOA did not greatly impact the observed geographic patterns of risk as compared to the referent models. However, odds of all cancers (Fig. 1A) and other solid tumors (Fig. 4A) increased after fully adjusting for PFOS or PFOA exposure and maternal characteristics.

When we examined the effects of the binary PFAS exposure variables in our models, we observed that PFOS was positively associated with all cancers among all participants in the exposure adjusted Model 2A [OR = 1.09, 95% CI: 0.99, 1.20], however this association fell short of statistical significance (Supplemental Table 5). Associations with PFOS were attenuated after adjustment for maternal characteristics in Model 2B [OR = 1.07, 95% CI: 0.97, 1.18] (Table 2). PFOS was significantly positively associated with overall solid tumors [OR = 1.14, 95% CI: 1.00, 1.31] in the exposure adjusted model, however the association fell short of significance in the fully adjusted model [OR = 1.12, 95% CI: 0.98, 1.29]. PFOS was also associated with higher odds of brain tumors [OR = 1.15, 95% CI: 0.96, 1.37] in fully adjusted models, however this association was not statistically significant. Odds of neuroblastoma [OR = 1.57, 95% CI:1.12, 2.20] and retinoblastoma [OR = 1.84, 95% CI: 1.21, 2.80] were associated with PFOS exposure and remained significant in fully adjusted models [OR = 1.51, 95% CI: 1.07, 2.13 and OR = 1.83, 95% CI: 1.19, 2.79, respectively]. Non-significant inverse associations were observed in fully adjusted models between PFOS exposure and nephroblastoma [OR = 0.79, 95% CI:0.50, 1.25] and leukemias [OR = 0.97, 95% CI: 0.83, 1.13].

**Table 2:** Effect estimates for associations between PFOS and PFOA exposures and childhoods diagnosed 2000-2021: Fully adjusted models^a^.

		PFOS			PFOA		
	*n*	OR	95% CI	*p*-value	OR	95% CI	*p*-value
All Cancers (Fig. 1)							
Overall	6448	1.07	0.97, 1.18	0.169	1.00	0.89, 1.13	0.950
US	3362	1.11	0.97, 1.27	0.130	1.04	0.88, 1.22	0.656
Mexico	1687	0.98	0.83, 1.17	0.859	0.98	0.78, 1.23	0.862
All Leukemias (Fig. 2)							
Overall	2070	0.97	0.83, 1.13	0.703	0.93	0.76, 1.12	0.431
US	1028	1.03	0.83, 1.28	0.774	0.99	0.76, 1.28	0.914
Mexico	603	0.88	0.67, 1.15	0.345	0.96	0.67, 1.38	0.839
Lymphoid Leukemias (Supplemental Fig. 2)							
Overall	1670	0.92	0.77, 1.09	0.326	0.93	0.75, 1.15	0.506
US	838	1.02	0.80, 1.30	0.866	1.04	0.78, 1.38	0.802
Mexico	497	0.83	0.61, 1.12	0.223	1.02	0.69, 1.51	0.914
Acute Myeloid Leukemia (Supplemental Fig. 3)							
Overall	305	1.19	0.82, 1.72	0.362	0.80	0.49, 1.30	0.362
US	141	1.03	0.59, 1.80	0.925	0.82	0.41, 1.65	0.582
Mexico	85	1.11	0.59, 2.08	0.757	0.61	0.23, 1.57	0.301
All Lymphomas (Supplemental Fig. 4A-C)							
Overall	691	1.12	0.89, 1.42	0.331	1.05	0.78, 1.43	0.744
US	362	1.12	0.80, 1.55	0.516	0.99	0.65, 1.52	0.978
Mexico	179	0.92	0.61, 1.37	0.670	0.79	0.45, 1.40	0.424
Non-Hodgkin Lymphoma (Supplemental Fig. 4D)							
Overall	247	1.03	0.71, 1.49	0.869	0.92	0.57, 1.51	0.752
Brain Tumors (Fig. 3)							
Overall	1301	1.15	0.96, 1.37	0.142	0.95	0.76, 1.19	0.676
US	748	1.16	0.91, 1.47	0.241	0.93	0.69, 1.26	0.646
Mexico	282	1.16	0.82, 1.65	0.400	1.16	0.74, 1.81	0.510
Other Solid Tumors (Fig. 4)							
Overall	2341	1.12	0.98, 1.29	0.109	1.09	0.92, 1.29	0.303
US	1197	1.17	0.96, 1.42	0.117	1.16	0.93, 1.46	0.195
Mexico	611	1.01	0.79, 1.29	0.960	0.99	0.72, 1.36	0.954
Neuroblastoma (Supplemental Fig. 5A)							
Overall	316	1.51	1.07, 2.13	0.019	1.17	0.76, 1.78	0.478
Retinoblastoma (Supplemental Fig. 5B)							
Overall	180	1.84	1.19, 2.79	0.006	1.85	1.14, 3.01	0.013
Nephroblastoma (Supplemental Fig. 5C)							
Overall	255	0.79	0.50, 1.25	0.316	0.99	0.59, 1.67	0.976

Abbreviations: Odds ratios (OR) and 95% confidence intervals (CI).

^a^Models adjusted for location, child sex, child age, and child’s birth year, maternal age, maternal, maternal birthplace, maternal race and ethnicity, and insurance provider; span size is 0.20 for all models.

PFOA was associated with an increased risk of solid tumors in the exposure adjusted Model 3A [OR = 1.12, 95% CI: 0.95, 1.32] (Supplemental Table 5) and these results were attenuated in the fully adjusted Model 3B [OR = 1.09, 95% CI: 0.92, 1.29] (Table 2), with neither result being statistically significant. PFOA was significantly associated with retinoblastoma [OR = 1.85, 95% CI: 1.14, 3.01] in the fully adjusted model. Non-significant inverse associations were observed in fully adjusted models between PFOA exposure and non-Hodgkin lymphoma [OR = 0.92, 95% CI: 0.57, 1.51] and leukemias [OR = 0.93, 95% CI: 0.76, 1.12].

### Children of US-born mothers

Spatial risk patterns of childhood cancer varied across cancer groups and within cancer groups for children of US-born mothers. In referent models (Model 1), child’s birth residence was statistically associated with all cancers, all leukemias, lymphoid leukemias, lymphomas, brain tumors, and other solid tumors, but not for acute myeloid leukemia (Supplemental Table 3). Increased risk was seen in areas served by Orange County water districts with reported levels of PFOS or PFOA for all cancers, all leukemias, lymphoid leukemias, lymphomas, and other solid tumors (Supplemental Table 4), however these areas were no longer present in fully adjusted models (Models 2B and 3B) of leukemias, lymphoid leukemias, and other solid tumors. Areas of decreased risk were seen in areas served by Los Angeles County water districts with reported levels of PFOS or PFOA for all cancers, all leukemias, lymphoid leukemias, and brain tumors, however these were no longer present in fully adjusted models of all cancers. Adjustment for PFOS (Model 2A) reduced the size and magnitude of areas of increased cancer risk as compared to referent models in analyses for risk of all cancers (Fig. 1B) and leukemias (Fig. 2B). Areas of increased risk were smaller in fully adjusted models for lymphomas (Supplemental Fig. 4B). The risk of other solid tumors was no longer significantly associated with location after adjustment for PFOS or PFOA in Model 2A and Model 3A (Fig. 4B). However, further adjustment for maternal characteristics in Model 2B and Model 3B, particularly race and ethnicity, increased the risk associated with location for other solid tumors, a pattern of reverse confounding that was not observed with any other cancer groups (Fig. 4B).

For the effect of PFAS exposures on childhood cancer, odds ratios associated with PFOS exposure were increased, however not statistically significant in both exposure adjusted Models 2A and 3A and fully adjusted Models 2B and 3B (Table 2, Supplemental Table 5). PFOS exposure was associated with an increased risk of all cancers in the exposure adjusted Model 2A [OR = 1.12, 95% CI: 0.98, 1.29] and in the fully adjusted Model 2B [OR = 1.11, 95% CI: 0.97, 1.27], however associations fell short of statistical significance. The association between other solid tumors and PFOS exposure was also elevated [OR = 1.19, 95% CI: 0.98, 1.44]. Associations between PFOA exposure and the cancer groups were not statistically significant and generally null or inversely associated.

### Children of Mexico-born mothers

The effect of location on childhood cancer also varied across cancer groups for children of Mexico-born mothers. In referent models (Model 1), child’s birth residence was statistically associated with all cancers, all leukemias, lymphoid leukemias, lymphomas and other solid tumors, but not for acute myeloid leukemia and brain tumors (Supplemental Table 3). Areas of increased risk were collocated with water districts in Los Angeles County with reported PFOS or PFOA for all cancers and lymphomas, and with Orange County water districts for all cancers, leukemias, lymphoid leukemias, lymphomas, and other solid tumors (Supplemental Table 4). Areas of decreased risk were seen in locations served by the Orange County water districts for leukemias and lymphoid leukemias. Adjustment for PFOS/PFOA (Models 2A and 3A) did not appear to explain the spatial pattern of cancer risks (Figs. 1–4C and Supplemental Figs. 2–4C) except for other solid tumors; the area of increased risk was no longer present after adjusting for PFOA (Model 3A).

Associations between PFOS/PFOA exposure and cancer groups were generally null or inversely associated. PFOS exposure was inversely associated with odds of leukemias [OR = 0.88, 95% CI: 0.67, 1.15] and lymphoid leukemias [OR = 0.83, 95% CI: 0.61, 1.12], and PFOA exposure was inversely associated with odds of lymphomas [OR = 0.79, 95% CI: 0.45, 1.40] in fully adjusted models (Table 2), however these associations were not statistically significant.

## Discussion

We observed areas of Los Angeles and Orange Counties with increased risks of childhood cancer, and child’s birth address remained a significant risk factor after adjustment for PFOS or PFOA exposure and maternal characteristics in the majority of our analyses, suggesting that those factors only marginally contributed to the excess spatial risk, and that other risk factors related to location remain unaccounted for. While there was some geographic overlap between statistically significant areas of childhood cancer risks and PFOS/PFOA contaminated water districts in referent models, only adjustment for PFOS exposure explained some of the spatial risk of cancers among children of US-born mothers, but not among those born to Mexico-born mothers. Increased odds of childhood cancers were associated with PFOS exposure, with neuroblastoma and retinoblastoma reaching statistical significance, while the odds of childhood cancers, with the exception of retinoblastoma, were null with respect to PFOA exposure.

Adjusting for PFOS/PFOA in drinking water and maternal characteristics, which are known to vary geographically, did not fully explain the underlying spatial variation in childhood cancer presented in the referent maps. For brain tumors, overall lymphomas, and lymphomas among children of US-born mothers, the effect of location was attenuated in fully adjusted models compared to models adjusting only for PFOS or PFOA. This is likely due to the clustering of socioeconomic factors in the study area that are risk factors for childhood cancer. Areas in northern Orange County and South Los Angeles experience concentrated poverty and have a majority population of people of color [36, 37]. Residents also experience some of the greatest income inequality of any United States metro region and are impacted by racial wage gaps. The odds of all cancers, solid tumors, and solid tumors among children of US-born mothers increased after fully adjusting for PFOS or PFOA exposure and maternal characteristics, suggesting negative confounding (i.e., location has a stronger effect in Models 2B and 3B after adjusting for maternal characteristics compared to Models 2A and 3A). For certain cancers, maternal characteristics may better explain the observed geographic risk pattern in referent models other than our estimated drinking water PFOS/PFOA exposures. Our observation that areas of increased risk remain after full adjustment suggest the potential for unmeasured spatial confounders.

Other studies of childhood cancer have studied associations with PFAS. PFOS was also associated with acute myeloid leukemia and neuroblastoma in our previous study of children born in California [23]; the participants from Los Angeles and Orange Counties in the current analyses are a subset of that state-wide population. However, all brain tumors and nephroblastoma were null in association with PFOS in our previous state-wide study, whereas we observed increased risks associated with PFOS in the present study limited to Southern California counties. The current analyses also found associations with PFOS and PFOA and retinoblastoma among Los Angeles and Orange County children, consistent with the results of previous work [27] with California cases and controls born between 1983 and 2011 (approximately 40% of their study population was born during years included in our study period), however other studies did not find associations with PFOA [23, 27]. Our results for lymphoid leukemias are consistent with other studies in California (2000-2015) and Finland (1986-2010) reporting no relationship with serum PFOS overall [23, 28], although the Finnish study reported a positive associations for pregnancy samples collected in 1986-1995, when PFOS levels were the highest [28].

Retinoblastoma and neuroblastoma, cancers that develop in nerve tissue, were significantly associated with PFAS in drinking water. Other case control studies have linked these tumors to environmental exposures, including air pollution, pesticides, residence in industrial or urban areas, or parental occupational exposures [6, 38–44]. Mechanistic evidence for the carcinogenicity of PFAS in nerve tissue is lacking, however there is some mechanistic evidence for PFAS exposure and other tumor types. PFAS were correlated with Ki-67 expression in studies of glioma and thyroid cancer [45, 46], and this protein associated with cell proliferation has also been identified as an indicator of neuroblastoma prognosis [47]. Future research is needed to further understand the mechanistic pathways by which PFAS exposure may contribute to cancer development in nerve tissue.

As compared to referent models, adjustment for PFOS in public water did not greatly affect the patterns of spatial variation in cancer risk seen among children of Mexico-born mothers, but it often attenuated the spatial patterns observed for children of US-born mothers (Fig. 1). This high-risk area is located in the same area as the PFAS-contaminated water districts (Supplemental Table 4). According to the National Health and Nutrition Examination Survey (2007-2014), Hispanic adults (including Mexican-Americans) were less likely to consume tap water than Non-Hispanic White adults [48]. Differing water consumption habits between US-born and Mexico-born mothers could explain the observed differences in their associations between PFOS-contaminated drinking water and cancer risk in locations in Los Angeles and Orange Counties.

The result of this study is informative, but should be considered exploratory given that the exposure assessment is limited to a narrow timeframe of public water measurements, without information on actual water consumption behaviors. Historical estimates of PFAS in these water systems throughout recent decades are not yet available, so the entire 3 years of UCMR3 sampling data were used to define drinking water exposure estimates for 22 years of diagnosis. The UCMR3 also had relatively high minimum reporting levels, causing water systems with lower levels of PFAS contamination to remain unreported. Preliminary data from UCMR5 suggest that the presence of PFAS in public water supplies is much more common than suggested by the UCMR3 data [22].

There are several strengths of this study. We were able to obtain a large sample size of childhood cancers, a rare disease, in a diverse area of Southern California that exhibits socioeconomic disparities. By using cancer registry data, impacts of selection bias are minimal due to the high reporting rate. This dataset also contained individual-level variables for location and maternal characteristics, allowing better characterization and confounding control for known and potential risk factors than studies using aggregated incidence and covariate data. By including residential location at birth as a covariate in GAMs, we were able to directly model the impact of location on cancer risk among our Los Angeles and Orange County population and analyze how adjustment for PFOS/PFOA drinking water exposure and other socioeconomic indicators contributed to the observed spatial variation of risk. However, there are other environmental exposures (e.g. air pollution) that are prevalent in Southern California and associated with childhood cancers, including brain tumors and lymphomas, that remain unaccounted for in this study. Our analysis was limited in its use of multiple testing for each cancer group, which may contribute to finding false positive associations. We were further limited in assessing independent effects of PFOS and PFOA due to the correlation between detects in water systems. We also used dichotomous exposure variables based on UCMR3 data, which had a high minimum reporting level so water districts with detectable but non-reported concentrations of PFOS/PFOA may have been misclassified. Additionally, the exposure potential during prenatal and postnatal periods was not able to be defined, as information regarding years spent at the birth residences was not available. Our analyses were also limited to confounders available in the CALSEC dataset, which did not include family history of cancer or household income. While we were able to use health insurance type and maternal education as a proxy for individual-level SES, there may be residual or unmeasured confounding in our analyses. Additionally, SES itself is a proxy for other risk factors for childhood cancer, including diet, infection, and other parental occupational exposures [49].

## Conclusion

PFOS and PFOA contamination of drinking water was associated with the risk of some childhood cancers in Los Angeles and Orange Counties, but PFOS and PFOA did not fully explain the significant geographic variation. The contribution of PFOS exposures to the spatial patterns of cancer risk was stronger among children of US-born mothers residing in areas served by PFAS-contaminated water districts compared to Mexico-born mothers, which may reflect differences in tap water consumption. Further research is needed to determine appropriate policy interventions in areas of increased risk.

## Supplementary information

Supplemental information

Supplemental table3,4,5

## Data Availability

UCMR3 data and water district shapefiles are publicly available at https://www.epa.gov/dwucmr/occurrence-data-unregulated-contaminant-monitoring-rule#3 and https://gis.data.ca.gov/datasets/45d26a15b96346f1816d8fe187f8570d_0/about, respectively. The CALSEC dataset analyzed during the current study is not publicly available due to the data use agreement at the California Department of Public Health and the California Cancer Registry.
