# Supplementary material for: Spatial analysis of residential location at birth, PFAS in public water, and childhood cancers in Southern California (2000–2019)
Source: J Expo Sci Environ Epidemiol. 2026 Mar 5;36(4):629–39. doi: 10.1038/s41370-026-00850-1 (PMC13032745; doi:10.1038/s41370-026-00850-1)
Supplement: Supplementary file 1 — Supplemental information [file 41370_2026_850_MOESM1_ESM.pdf]

Supplemental Information:

Spatial analysis of residential location at birth, PFAS in public water, and childhood cancers in Southern California (2000-2019)

Author names and affiliations: Natalie R. Binczewski[a], Libby M. Morimoto, PhD[b], Joseph L. Wiemels, PhD[c], David B. Richardson[a], Scott M. Bartell[a], Catherine Metayer, MD, PhD[b], Veronica M. Vieira, DSc[a]

[a] Department of Environmental and Occupational Health, Joe C. Wen School of Population & Public Health, University of California, Irvine, CA, 92617, USA (nbinczew@uci.edu and vvieira@uci.edu)

[b] Division of Epidemiology, School of Public Health, University of California, Berkeley, CA, 94704, USA. (libbym@berkeley.edu and cmetayer@berkeley.edu)

[c] Center for Genetic Epidemiology, Department of Population and Public Health Sciences, University of Southern California Keck School of Medicine, Los Angeles, CA 90033, USA. (wiemels@usc.edu)

Summary: 5 Tables, 5 Figures. Supplemental Table 3, Supplemental Table 4, and Supplemental Table 5 included as an Excel file.

Supplemental Table 1. Cancer groups as defined by International Classification of Childhood Cancer (ICCC), Third edition (IARC 2017).

Cancer Groups	ICCC Site Group Classification[a]
**Leukemias**	I
**Lymphoid leukemias**	I a
**Acute myeloid leukemias**	I b
**Lymphomas**	II
**Non-Hodgkin lymphoma**	II b
**Brain tumors**	III
**Other solid tumors**	IV - XII and not classified
**Neuroblastoma**	IV a
**Retinoblastoma**	V
**Nephroblastoma**	VI a 1

[a] ICCC classifications are coded into main groups (roman numerals), subgroups (lowercase letters), and divisions (numbers)

Supplemental Table 2. Map odds ratio (OR) ranges (lower, upper) for location in GAMs using span size optimized by minimizing the AIC.

Cancer Groups	n cases	span	OR	p-value
All Cancers	6448	0.20	0.77-1.40	< 0.001
All Leukemias	2070	0.80	0.84-1.14	0.134
Lymphoid Leukemias	1670	0.80	0.81-1.18	0.071
Acute Myeloid Leukemia	305	0.95	0.86-1.18	0.614
Lymphomas	691	0.25	0.28-1.45	0.001
Non-Hodgkin Lymphoma	247	0.75	0.72-1.49	0.09
Brain Tumors	1301	0.10	0.42-3.94	< 0.001
Other Solid Tumors	2341	0.15	0.67-1.40	< 0.001
Neuroblastoma	316	0.95	0.86-1.12	0.822
Retinoblastoma	180	0.80	0.52-1.32	0.134
Nephroblastoma	255	0.95	0.79-1.12	0.797

Supplemental Figure 1. Map of the study area. CALSEC cases and controls with Los Angeles and Orange County zip codes at birth (2000-2019). A) Maps of Los Angeles and Orange Counties with city boundaries. B) Water district service area boundaries in Los Angeles and Orange County

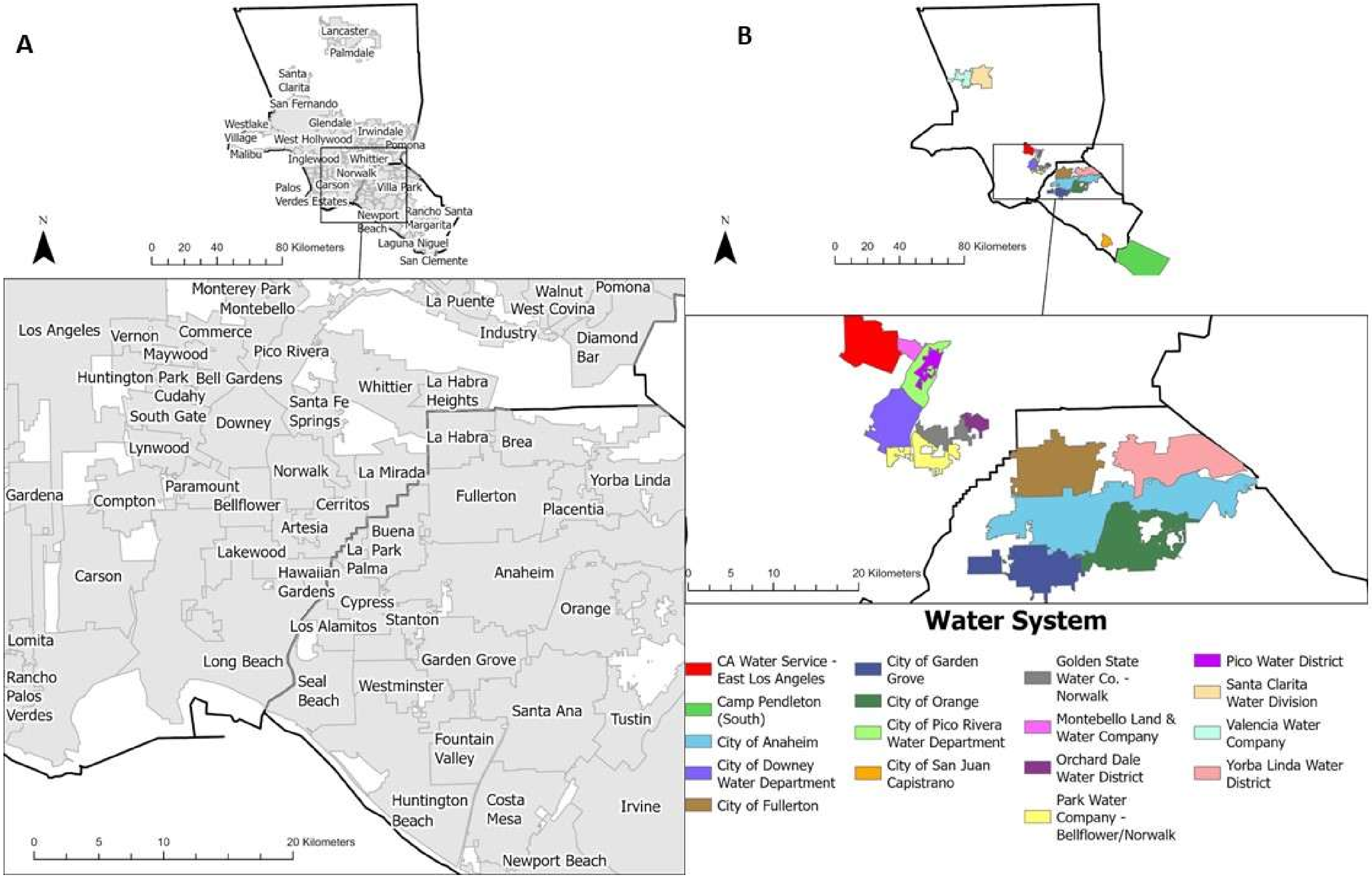

Supplemental Figure 2. Geographic patterns of lymphoid leukemias in Los Angeles and Orange Counties, California, 2000-2021. Areas of statistically significant increased or decreased risk are outlined in black. A) Models for lymphoid leukemia cases (n=1670) and controls (n=13044). B) Models for lymphoid leukemia cases (n=838) and controls (n=6338) born to US-born mothers. C) Models for lymphoid leukemia cases (n=497) and controls (n=3707) born to Mexico (MX)-born mothers.

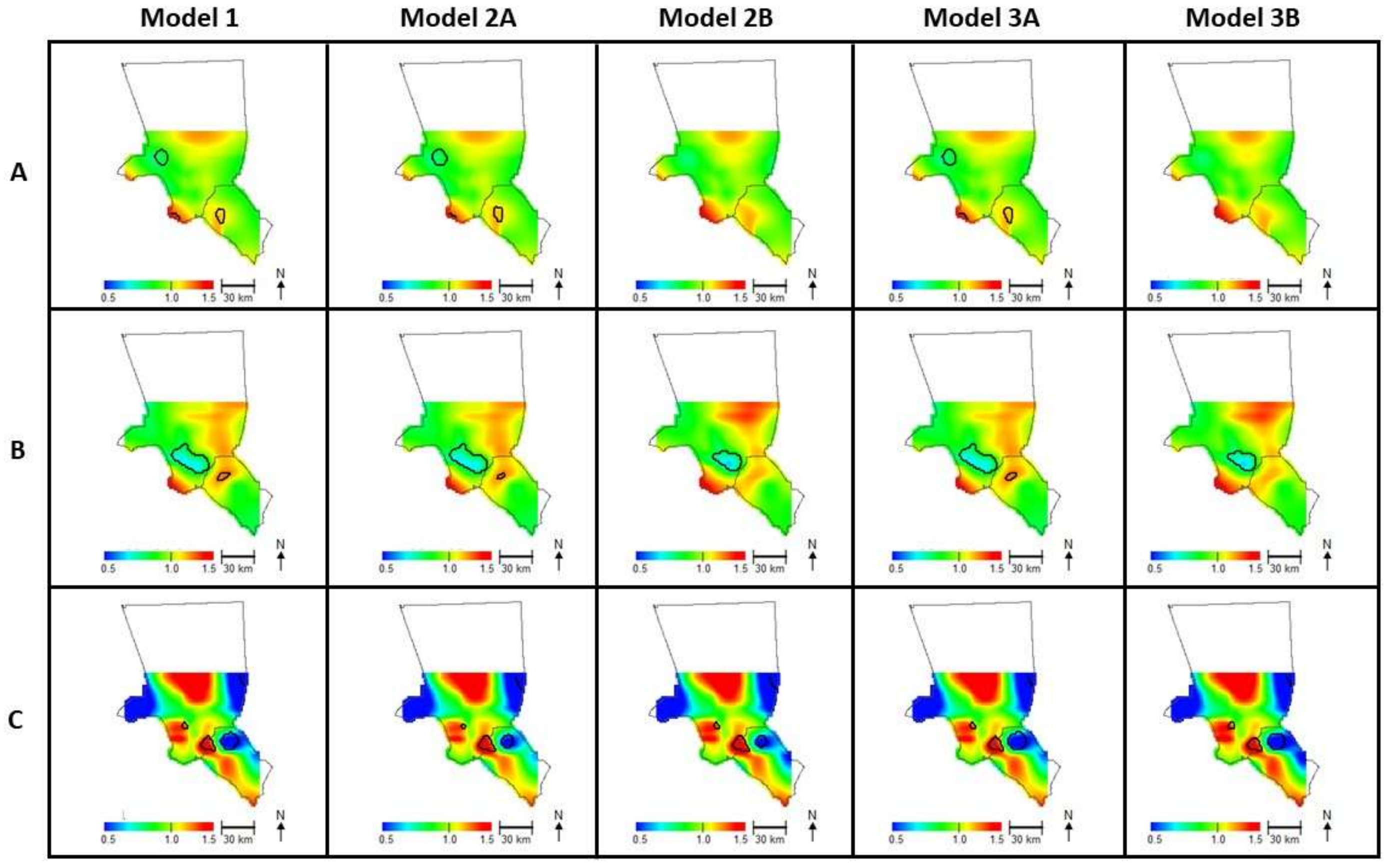

Model 1 adjusts for location, child sex, child age, and child's birth year.
Model 2A adjusts for PFOS exposure, in addition to adjustments in Model 1.
Model 2B adjusts for maternal age, maternal, maternal birthplace, maternal race and ethnicity, and insurance provider, in addition to adjustments in Model 2A.
Model 3A adjusts for PFOA exposure, in addition to adjustments in Model 1.
Model 3B adjusts for maternal age, maternal, maternal birthplace, maternal race and ethnicity, and insurance provider, in addition to adjustments in Model 3A.

Supplemental Figure 3. Geographic patterns of acute myeloid leukemias in Los Angeles and Orange Counties, California, 2000-2021. Areas of statistically significant increased or decreased risk are outlined in black. A) Models for acute myeloid leukemias cases (n=305) and controls (n=13044). B) Models for acute myeloid leukemias cases (n=141) and controls (n=6338) born to US-born mothers. C) Models for acute myeloid leukemias cases (n=85) and controls (n=3707) born to Mexico (MX)-born mothers.

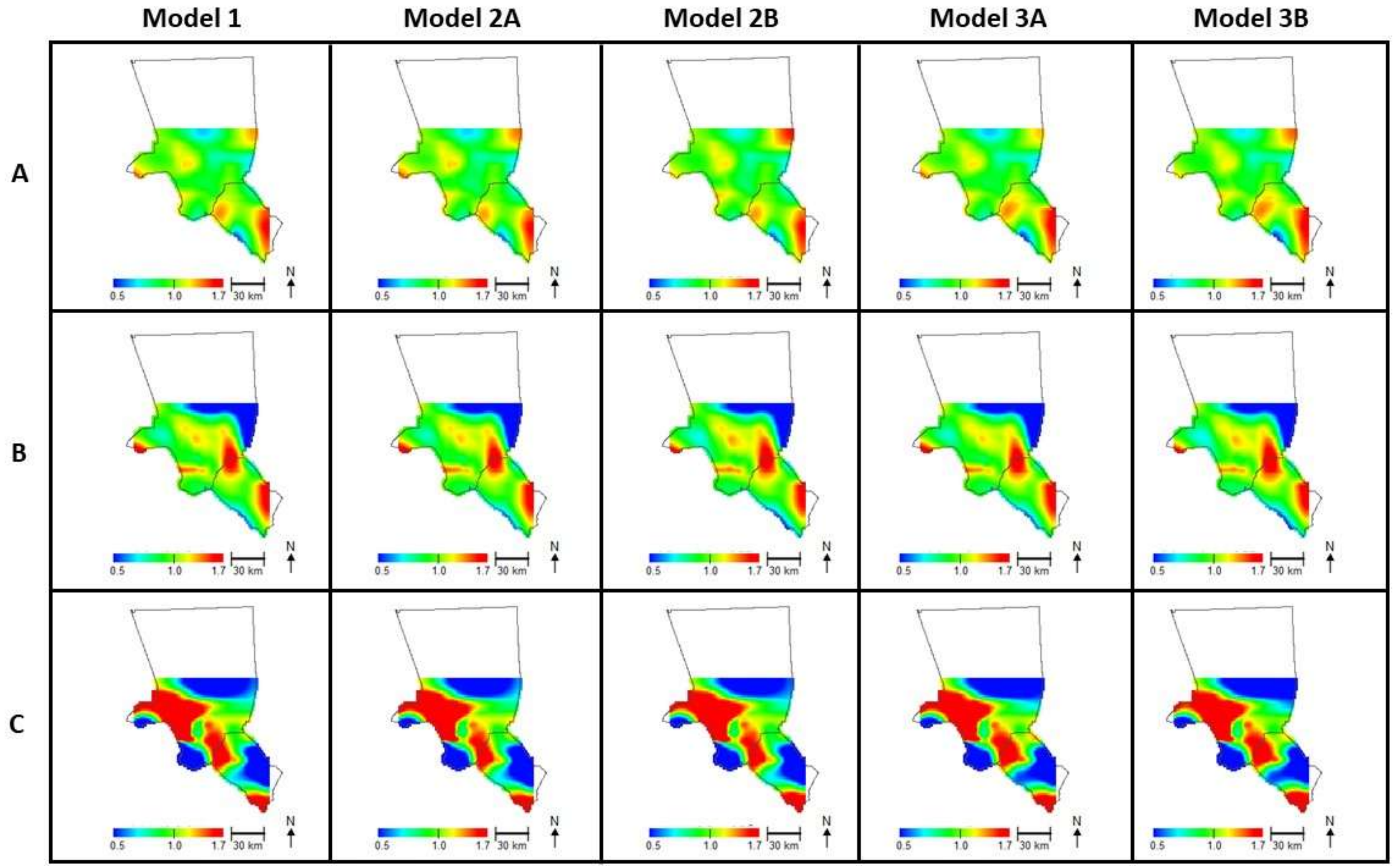

Model 1 adjusts for location, child sex, child age, and child's birth year.
Model 2A adjusts for PFOS exposure, in addition to adjustments in Model 1.
Model 2B adjusts for maternal age, maternal, maternal birthplace, maternal race and ethnicity, and insurance provider, in addition to adjustments in Model 2A.
Model 3A adjusts for PFOA exposure, in addition to adjustments in Model 1.
Model 3B adjusts for maternal age, maternal, maternal birthplace, maternal race and ethnicity, and insurance provider, in addition to adjustments in Model 3A.

Supplemental Figure 4. Geographic patterns of all lymphomas in Los Angeles and Orange Counties, California, 2000-2021. Areas of statistically significant increased or decreased risk are outlined in black. A) Models for lymphoma cases (n=691) and controls (n=13044). B) Models for lymphoma cases (n=362) and controls (n=6338) born to US-born mothers. C) Models for lymphoma cases (n=179) and controls (n=3707) born to Mexico (MX)-born mothers. D) Models for non-Hodgkin lymphoma cases (n=247) and controls (n=13044).

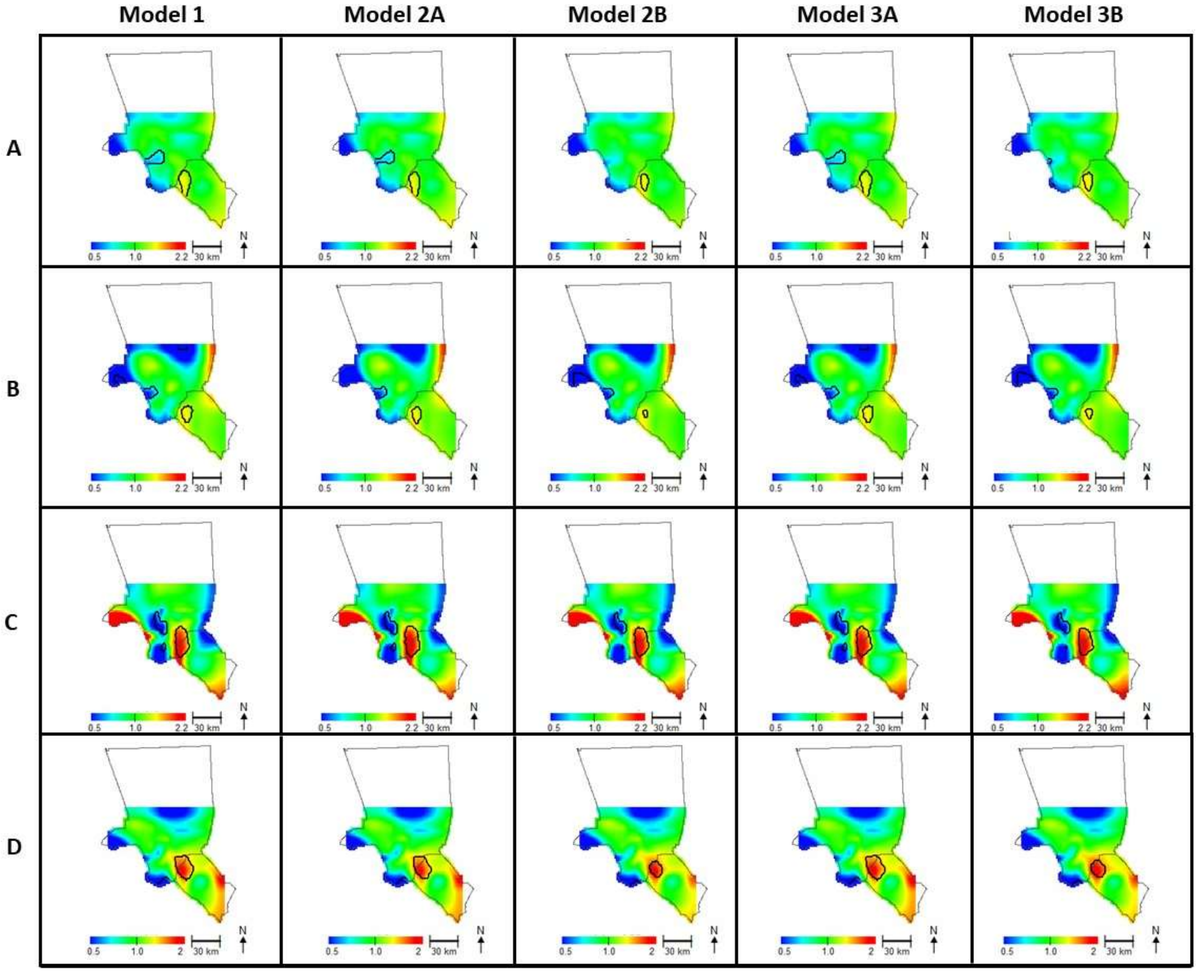

Model 1 adjusts for location, child sex, child age, and child’s birth year.
Model 2A adjusts for PFOS exposure, in addition to adjustments in Model 1.
Model 2B adjusts for maternal age, maternal, maternal birthplace, maternal race and ethnicity, and insurance provider, in addition to adjustments in Model 2A.
Model 3A adjusts for PFOA exposure, in addition to adjustments in Model 1.
Model 3B adjusts for maternal age, maternal, maternal birthplace, maternal race and ethnicity, and insurance provider, in addition to adjustments in Model 3A.

Supplemental Figure 5. Geographic patterns of other cancer types in Los Angeles and Orange Counties, California, 2000-2021. Areas of statistically significant increased or decreased risk are outlined in black. A) Models for neuroblastoma cancer cases (n=316) and controls (n=13044). B) Models for nephroblastoma cancer cases (n=255) and controls (n=13044) born to US-born mothers. C) Models for retinoblastoma cancer cases (n=180) and controls (n=13044) born to Mexico (MX)-born mothers.

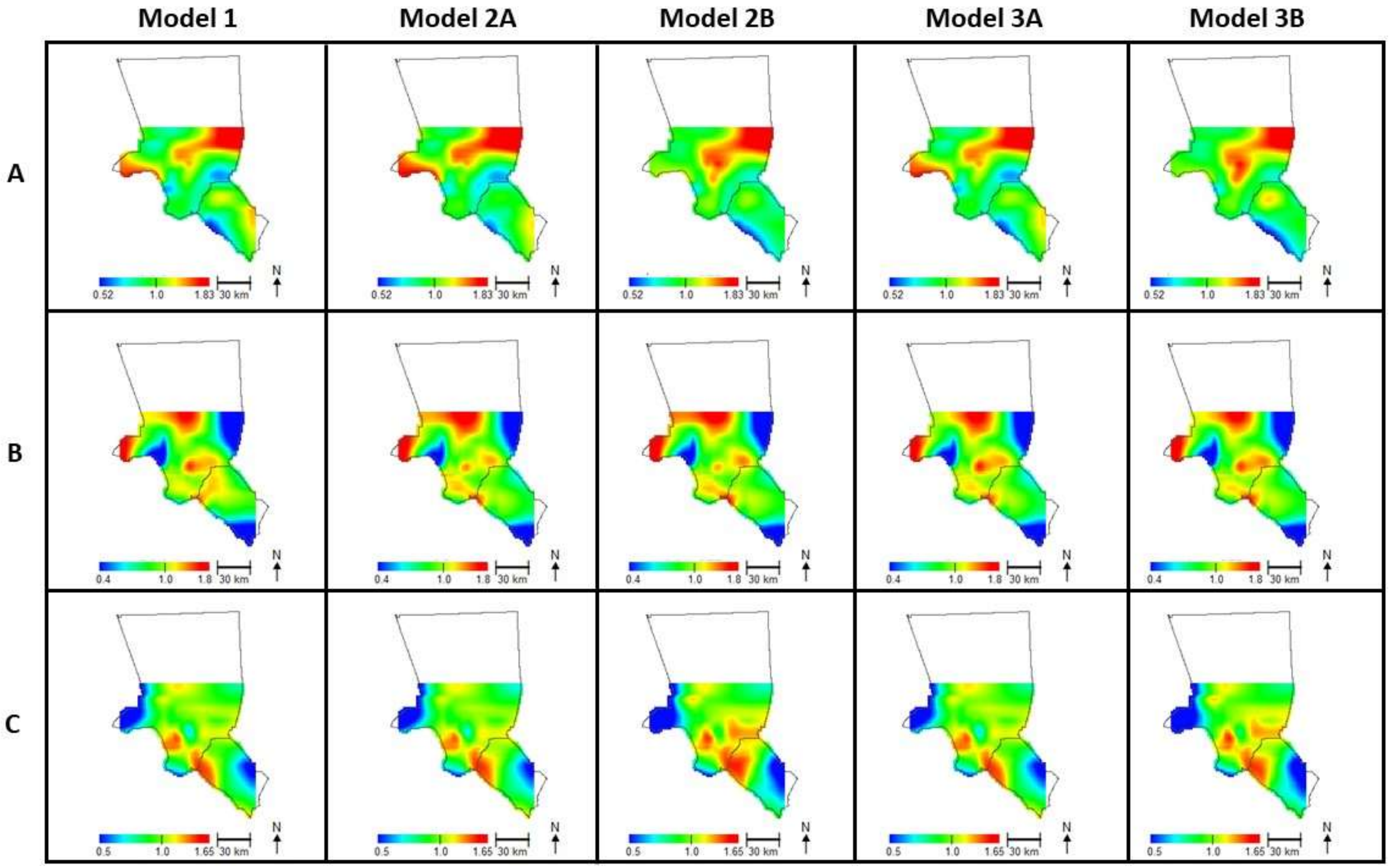

Model 1 adjusts for location, child sex, child age, and child's birth year.
Model 2A adjusts for PFOS exposure, in addition to adjustments in Model 1.
Model 2B adjusts for maternal age, maternal, maternal birthplace, maternal race and ethnicity, and insurance provider, in addition to adjustments in Model 2A.
Model 3A adjusts for PFOA exposure, in addition to adjustments in Model 1.
Model 3B adjusts for maternal age, maternal, maternal birthplace, maternal race and ethnicity, and insurance provider, in addition to adjustments in Model 3A.
